# Reelin Regulates Neuronal Excitability through Striatal-Enriched Protein Tyrosine Phosphatase (STEP_61_) and Calcium Permeable AMPARs in an NMDAR-Dependent Manner

**DOI:** 10.1523/JNEUROSCI.0388-21.2021

**Published:** 2021-09-01

**Authors:** Murat S. Durakoglugil, Catherine R. Wasser, Connie H. Wong, Theresa Pohlkamp, Xunde Xian, Courtney Lane-Donovan, Katja Fritschle, Lea Naestle, Joachim Herz

**Affiliations:** ^1^Department of Molecular Genetics; ^2^Center for Translational Neurodegeneration Research; ^3^Departments of Neuroscience and; ^4^Neurology and Neurotherapeutics, University of Texas Southwestern Medical Center, Dallas, Texas 75390; ^5^Institute of Cardiovascular Sciences and Key Laboratory of Molecular Cardiovascular Sciences, Ministry of Education, Peking University, Beijing 100871, China; ^6^Weill Institute for Neurosciences, University of California San Francisco, San Francisco, California 94158; ^7^Technical University of Munich, 80333 Munich, Germany; ^8^Ludwig-Maximilians University of Munich, 80539, Munich, Germany

**Keywords:** AMPA receptor, ApoE, NMDA receptor, Reelin, STEP, synaptic plasticity

## Abstract

Alzheimer's disease (AD) is a progressive neurodegenerative disease marked by the accumulation of amyloid-β (Aβ) plaques and neurofibrillary tangles. Aβ oligomers cause synaptic dysfunction early in AD by enhancing long-term depression (LTD; a paradigm for forgetfulness) via metabotropic glutamate receptor (mGluR)-dependent regulation of striatal-enriched tyrosine phosphatase (STEP_61_). Reelin is a neuromodulator that signals through ApoE (apolipoprotein E) receptors to protect the synapse against Aβ toxicity (Durakoglugil et al., 2009) Reelin signaling is impaired by ApoE4, the most important genetic risk factor for AD, and Aβ-oligomers activate metabotropic glutamate receptors (Renner et al., 2010). We therefore asked whether Reelin might also affect mGluR-LTD. To this end, we induced chemical mGluR-LTD using DHPG (Dihydroxyphenylglycine), a selective mGluR5 agonist. We found that exogenous Reelin reduces the DHPG-induced increase in STEP_61_, prevents the dephosphorylation of GluA2, and concomitantly blocks mGluR-mediated LTD. By contrast, Reelin deficiency increased expression of Ca^2+^-permeable GluA2-lacking AMPA receptors along with higher STEP_61_ levels, resulting in occlusion of DHPG-induced LTD in hippocampal CA1 neurons. We propose a model in which Reelin modulates local protein synthesis as well as AMPA receptor subunit composition through modulation of mGluR-mediated signaling with implications for memory consolidation or neurodegeneration.

**SIGNIFICANCE STATEMENT** Reelin is an important neuromodulator, which in the adult brain controls synaptic plasticity and protects against neurodegeneration. Amyloid-β has been shown to use mGluRs to induce synaptic depression through endocytosis of NMDA and AMPA receptors, a mechanism referred to as LTD, a paradigm of forgetfulness. Our results show that Reelin regulates the phosphatase STEP, which plays an important role in neurodegeneration, as well as the expression of calcium-permeable AMPA receptors, which play a role in memory formation. These data suggest that Reelin uses mGluR LTD pathways to regulate memory formation as well as neurodegeneration.

## Introduction

Alzheimer's disease (AD) is a socioeconomic burden of sweeping proportions. Dysregulation of synaptic function occurs in AD before overt clinical manifestation. One of the main characteristics of AD are amyloid-β (Aβ) plaques. Aβ oligomers cripple the synapses through multiple modes of action and hijack the core neuronal machinery leading to inhibition of long-term potentiation (LTP) and enhancement of long-term depression (LTD), paradigms for learning and memory or forgetfulness, respectively.

At excitatory synapses, glutamate binds to the ionotropic AMPA and NMDA receptors (AMPAR and NMDAR, respectively) as well as metabotropic glutamate receptors (mGluRs). AMPARs regulate fast synaptic transmission under resting conditions. NMDARs are coincidence detectors that flux Ca^2+^ on activation, which in turn regulates LTP through transcriptional mechanisms. In contrast, mGluR activation regulates LTD by modulating local synaptic mRNA translation. (Ebert and Greenberg, 2013).

AMPARs are tetramers consisting of combinations of four subunits: GluA1-4. GluA2 is critical for determining AMPAR function, and GluA2 depletion ablates mGluR-LTD starting at the age of 3 months in mice (Zhou et al., 2011; Cao et al., 2017; 2018). During LTD, one of the main translated proteins is striatal-enriched tyrosine phosphatase (STEP_61_). Phosphorylation of STEP_61_ regulates synaptic plasticity by dephosphorylation of the NMDAR subunit GluN2B and AMPAR subunit GluA2, which lead to the endocytosis of the receptors (Pelkey et al., 2002; Snyder et al., 2005; Zhang et al., 2008). Importantly, Aβ induces endocytosis of GluA2-containing AMPARs, mimicking and occluding mGluR-dependent LTD (Hsieh et al., 2006). In AD patients and mouse models, STEP_61_ levels are increased (Kurup et al., 2010). Blocking mGluR5 rescues cognitive impairment in an AD mouse model (Renner et al., 2010; Rammes et al., 2011; Chen et al., 2013). Importantly, the inhibition (Xu et al., 2014) or genetic knockdown (Zhang et al., 2011) of STEP_61_ ameliorates the behavioral and cognitive impairments seen in AD mouse models, implicating STEP_61_ as a downstream target of Aβ and mGluR5.

We have shown previously that the extracellular signaling protein Reelin, by binding to ApoE (apolipoprotein E) receptors, can antagonize Aβ at the synapse (Durakoglugil et al., 2009) and protect neurons against Aβ toxicity *in vivo* (Lane-Donovan et al., 2015). This Reelin protective effect is inhibited by ApoE4, the most important genetic risk factor for late-onset AD, as it impairs the recycling of Apoer2 and glutamate receptors (Chen et al., 2010; Xian et al., 2018). Reelin regulates the surface expression of both AMPARs (Qiu et al., 2006a; Qiu and Weeber, 2007) and NMDAR (Weeber et al., 2002; Chen et al., 2005; Durakoglugil et al., 2009) and is essential for the NMDAR subunit switch during development (Groc et al., 2007).

In this current study, we investigated whether Reelin can modulate mGluR-LTD by either applying exogenous Reelin on wild-type hippocampal slices or by using Reelin-deficient slices and determining STEP_61_ and AMPAR composition. We used a mouse model with induced genetic depletion of Reelin [conditional KO (cKO)], which has an architecturally normal adult brain (Lane-Donovan et al., 2015). Previously, we have shown that Reelin cKO mice have enhanced LTP, are hypersensitive to Aβ-induced synaptic suppression, and have profound memory and learning disabilities in the presence of low Aβ levels (Lane-Donovan et al., 2015), which can be reversed by treating the mice for 1 month with the g-secretase modulator NGP555 (Kounnas et al., 2017). Here, we now show that acute Reelin application reduces both mGluR-LTD and STEP_61_ levels. Reelin cKO mice, on the other hand, display increased STEP_61_ levels and increased expression of surface GluA2-lacking AMPARs, which results in the occlusion of mGluR-LTD. Overall, our data demonstrate that Reelin, through regulation of local protein synthesis, controls the assembly of AMPAR subunits on the synaptic surface, with implications for memory consolidation and neurodegeneration.

## Materials and Methods

### 

#### 

##### Animals

All mice were housed under a 12:12 light/dark cycle and fed a normal chow diet. All animals were killed by inhalation of isoflurane followed by decapitation according to strict regulations set by the National Institutes of Health Guide for the Care and Use of Laboratory Animals and the University of Texas Southwestern Animal Care and Use Committee. Pregnant Sprague Dawley rats were purchased from Charles River Laboratories. The Reelin conditional knock-out animals have been described previously (Lane-Donovan et al., 2015). Briefly, Exon1 of the *Reln* gene was flanked by loxP sites. Homozygous Reelin-floxed (*Reln^fl/fl^*) mice were then crossed with transgenic CAG-Cre^ERT2^ mice (Hayashi and McMahon, 2002). To induce conditional Reelin knockout, Cre-positive *Reln^fl/fl^* mice were injected with tamoxifen (Reelin cKO) as described (Lane-Donovan et al., 2015). As a control, Cre-negative *Reln^fl/fl^* mice were also injected with tamoxifen. For electrophysiological recordings, tamoxifen injections were performed at 4 weeks of age. For biochemical analysis, tamoxifen injections were performed at 8 weeks of age by daily intraperitoneal injections for 5 d with 135 mg/kg tamoxifen (Sigma) dissolved in sunflower oil and 10% ethanol. Electrophysiological recordings and biochemical analysis were performed at 3 or 6 months, as indicated. This gave us the advantage of testing the *in vivo* function of endogenous Reelin in adult mice after the end of the neuronal migration period for which Reelin is required (Herz and Chen, 2006).

##### Recombinant protein

Recombinant Reelin was secreted by 293HEK cells stably transfected with the mouse Reelin cDNA plasmid (pCrl). Reelin was purified from the media as described (Weeber et al., 2002).

##### *In vitro* electrophysiology (whole-cell patch-clamp recordings)

Cells were recorded from the CA1 region of 2- 3-month-old mice. The extracellular solution contained the following (in mm): 124 NaCl, 3 KCl, 1.25 NaH_2_PO_4_, 26 NaHCO_3_, 10 D-glucose, 2 CaCl_2_, 1 MgSO_4_. The intracellular solution contained the following (in mm): 130 CsMeSO_4_, 5 NaCl, 10 HEPES, 1 CaCl_2_, 4 MgCl_2_, 0.5 EGTA, four MgATP, 5 QX314, 0.3 GTP and 0.1 spermine (pH adjusted with CsOH). Spontaneous recordings of AMPAR currents were performed at room temperature (26°C) in the presence of the NMDAR antagonist d-AP5 (D-(-)−2-Amino-5-phosphonopentanoic acid; 50 µm) and GABA receptor antagonist picrotoxin (50 µm). Stimulus intensity was adjusted to evoke currents approximately one-third of the maximal current response, typically resulting in currents between 100 and 200 pA. Current voltage curves were obtained by plotting the current response at different holding potentials (−70 to +50 mV with 10 mV increments). The rectification index (RI) was calculated as the ratio of the average peak current responses at −70 mV to those at 50 mV. NASPM [1-Naphhtylacetyl spermine trihydrochloride; philanthotoxins (PhTX), 100 µm] was applied after a 20 min stable baseline for ∼20 min and washed out for at least 20 min.

##### Extracellular field recordings

Hippocampal slices were prepared from 3-month-old Reelin cKO mice and Cre-negative, tamoxifen-injected littermate controls. The brains were quickly removed and placed in cold high sucrose cutting solution containing the following (in mm): 110 sucrose, 60 NaCl, 3 KCl, 1.25 NaH_2_PO_4_, 28 NaHCO_3_, 0.5 CaCl_2_, 5 glucose, 0.6 Ascorbic acid, 7 MgSO_4_. Transverse sections of 400 µm were cut using a vibratome. Slices were then transferred into an incubation chamber containing 50% artificial CSF (aCSF) containing the following (in mm): 124 NaCl, 3 KCl, 1.25 NaH_2_PO_4_, 26 NaHCO_3_, 10 D-glucose, 2 CaCl_2_, 1 MgSO_4_) and 50% sucrose cutting solution. In the recording chamber, slices were perfused with aCSF only. For stimulation, concentric bipolar electrodes were used (catalog #CBBRC75, FHC) and placed into the stratum radiatum. Experiments were performed in the presence of d-AP5 (50 µm) and picrotoxin (10 µm). Twenty minutes after stable baseline, DHPG (100 µM) was applied for 10 minutes. Stimulus intensity was set at 40–60% of maximum response and delivered through an Isolated Pulse Stimulator (Model 2100, A-M. Systems). A custom-written program in LabVIEW 7.0 was used for recording and analysis of LTD experiments.

##### Pharmacological treatment of slices

**Figure 1.**
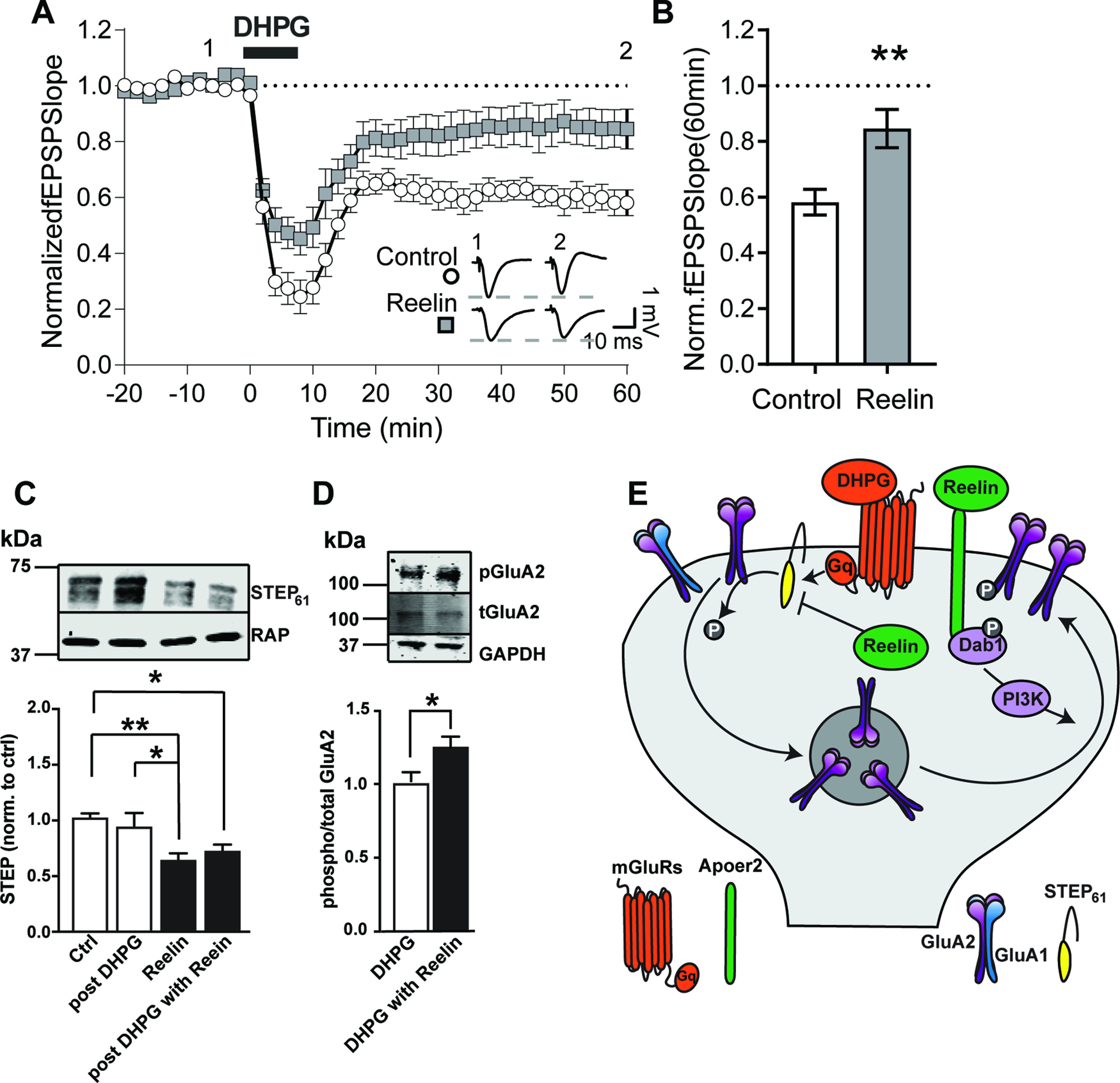
Reelin blocks DHPG-induced LTD through regulating STEP and phospho-GluA2 levels. ***A***, Field EPSPs were recorded from stratum radiatum in the presence of AP-5 (50 μm) and picrotoxin (10 μm). Mock or Reelin was added 10 min before recordings and was present throughout the experiment (−30 to +60). Stable baseline fEPSP was recorded for 20 min (−20 to 0 min) before DHPG application and normalized to 1. DHPG was applied for 10 min (0–10 min). Sample traces before and after (−10, +40 min) DHPG in the presence or absence of Reelin are shown (bottom right inset; 3-month-old mice; Control, *n* = 14 slices, *a* = 8 animals; Reelin, *n* = 11, *a* = 6). Two-way RM ANOVA: Reelin-int: *F*_(1,23)_ = 9.68, *p* = 0.0049. Sidak's *post hoc*: Control versus Reelin, 50–60 min: *p* = 0.0493-0.0016, time-int: *F*_(40,920)_ = 55.8, *p* < 0.0001; Sidak's *post hoc*: Control: −20 versus 60, *p* < 0.0001; Reelin: −20 versus 60 min, *p* = 0.3385. -int, -interaction. ***B***, Bar graph shows the fEPSP slope average of the last 10 min of recording from ***A*** (50–60 min). Unpaired Student's *t* test was performed on the average of control slices or Reelin-treated slices 50 min after DHPG treatment (Control *n* = 14, Reelin *n* = 11, *p* = 0.0031). ***C***, Exogenous Reelin decreases STEP_61_ in wild-type slice from 3-month-old mice treated with or without DHPG for 10 min followed by a washout (post-DHPG). Slices were oxygenated in aCSF containing picrotoxin and AP-5 in a six-well plate. Twenty minutes after pretreatment with or without Reelin, slices were moved to DHPG-containing wells for 10 min. DHPG was washed out, and slices were incubated for an additional 30 min with or without Reelin. Total STEP_61_ levels were analyzed by immunoblotting and quantified by averaging seven independent experiments from seven different mice (bar graph). ANOVA *F*_(3,24)_ = 5.591 *p* = 0.0047. Holm–Sidak multiple comparisons test shows a significant difference between Control versus Reelin (*p* = 0.0096) and Control versus post-DHPG with Reelin (*p* = 0.0482). All data are plotted as mean ± SEM. ***D***, Exogenous Reelin reverses DHPG-induced reduction of phospho-GluA2 (pGluA2) in rat primary neurons. Neurons were pretreated for 30 min in aCSF containing picrotoxin and AP-5. Reelin and/or DHPG was added for an additional hour before neurons were harvested for Western blot. The quantification of pGluA2/tGluA2 is shown in the bar graph (bottom; *n* = 4 independent cultures, *p* = 0.03, unpaired *t* test). All data are plotted as mean ± SEM. ***E***, Reelin activates SFK and stabilizes GluA2 on the cell surface, whereas STEP_61_ dephosphorylates and removes GluA2 from the surface. We propose a model in which Reelin blocks mGluR-dependent protein synthesis and LTD through STEP_61_. Error bars represent the standard error of the mean (SEM). (*p < 0.05, **p < 0.01).

Experiments were performed in six-well plates (see above, Extracellular field recordings). Briefly, 300 µm WT slices were continuously bubbled with 95% O_2_ and 5% CO_2_ in aCSF containing AP-5 and picrotoxin (Fig. 1*C*). Four of the slices obtained were incubated with 50 µm AP-5 and 10 µm picrotoxin; the other four were incubated with 5 nm Reelin with AP-5 and picrotoxin. Twenty minutes later, two of the slices from both the control group and the Reelin-treated group were incubated with 100 µm DHPG (AP-5+picrotoxin ± Reelin). Ten minutes later, slices were washed in either AP-5- and picrotoxin-containing aCSF or in AP5-, picrotoxin-, and Reelin-containing aCSF for 30 min, and then collected for biochemical analysis.

For the experiments on Reelin cKO (Cre-positive mice injected with tamoxifen) and Control (Cre-negative littermates injected with tamoxifen; see Fig. 5) slices were bubbled with 95% O_2_ and 5% CO_2_ in aCSF. Both Control and Reelin cKO slices were incubated in wells containing AP-5- and picrotoxin-containing aCSF for 1 h. Slices were collected for biochemical analysis (see below, Surface biotinylation and Western blotting).

##### Surface biotinylation

Biotinylation experiments were performed as described previously (Nosyreva and Huber, 2005). Slices were biotinylated on ice for 30 min using PBS buffer containing a nonpermeable biotin solution, 1.0 mg/ml sulfo-NHS-SS-biotin (Pierce). Slices were homogenized by forcing the suspension 15–20 times through a 23-gauge needle in 300 µl of ice-cold lysis buffer containing the following (in mm): 65 Tris-HCl pH 7.4, 150 NaCl, 1 EDTA, 0.5% deoxycholic acid, 1% Triton X-100, 0.1% SDS). Homogenate was spun down at 14,000 rpm for 15 min at 4°C. Protein concentration of the supernatant was determined using the Bio-Rad DC protein assay. Surface proteins were pulled down using neutravidin beads (Thermo Scientific). Beads were boiled for 20 min in 5× SDS sample buffer (250 mm Tris-HCl, pH 6.8), 500 mm DTT, 10% SDS, 0.5% bromphenol blue, 50% glycerol) at 90°C. For total protein, SDS sample buffer (1× final) was added to 10 µg of hippocampal lysate and incubated for 10 min at 90°C. The surface and total protein samples were separated on an 8% SDS-PAGE gel and transferred to a nitrocellulose membrane.

##### Western blotting

Six-month-old Reelin cKO mice were perfused, and brains were quickly isolated and flash frozen. Hippocampi were dissected in PBS with protease inhibitors and then lysed with lysis buffer containing the following (in mm): 0.1 ammonium molybdate, 2 EDTA, 2 sodium pervanadate, 50 sodium fluoride, 10 sodium pyrophosphate, 320 sucrose; 5× sample buffer was added to hippocampal lysate. Samples were boiled at 80°C for 10 min and then loaded on a Tris/Glycine gradient gel and transferred onto a PVDF membrane.

The surface and total protein samples were separated on an 8% SDS-PAGE gel and transferred to a nitrocellulose membrane. Surface and total levels of GluA subunits were determined with antibodies directed against GluA2/3 (catalog #07-598, Millipore), GluA1 (catalog #ab1504, Abcam), or GluA4 (catalog #3824S, Cell Signaling Technology). STEP_61_ of total fractions was visualized with an antibody directed against STEP (Cell Signaling Technology, 23e5) and GAPDH with anti GAPDH antibody (catalog #ab9485, Abcam). Protein levels were calculated by using the LI-COR Biosciences system. The ratio of surface to total protein levels was calculated after normalizing to GAPDH. Reelin knockdown was confirmed by probing with anti-Reelin antibody (G10; de Bergeyck et al., 1998).

##### Primary cortical neuronal cultures

Primary cortical neuronal cultures were prepared from rat (SD, Charles River Laboratories) at E18 as described previously (Chen et al., 2005). Neurons were cultured in Poly-D-Lysine-coated six-well plates (1 million/9 cm^2^) in the presence of neurobasal medium supplemented with B27, glutamine, and penicillin-streptomycin and incubated at 37°C with 5% CO_2_. Primary neurons were used at DIV 13–15 for experiments. Media was changed to aCSF 2 h before DHPG (100 µm) was applied with or without Reelin (5 nm) for 1 h in the presence of AP-5 (50 µm) and picrotoxin (10 µm).

##### Drugs

(RS)−3,5-DHPG, d-AP5, and spermine tetrahydochloride were purchased from R&D Systems. NASPM and picrotoxin were purchased from Sigma-Aldrich.

##### Statistical analysis

Data were expressed as the mean ± SEM and evaluated using two-tailed Student's *t* test for two groups with one variable tested and equal variances or one-way ANOVA with Dunnett's *post hoc* or Tukey's *post hoc* for multiple groups with only variable tested. The differences were considered significant at *p* < 0.05 (*p < 0.05, **p < 0.01, ***p < 0.001). Software used for data analysis was ImageJ (National Institutes of Health), LabVIEW 7.0 (National Instruments), Odyssey Imaging Systems (LI-COR Biosciences), Prism 7.0 (GraphPad Software).

## Results

### Reelin blocks DHPG-Induced LTD in hippocampal slices from wild-type mice

Synaptic plasticity is mediated by both LTD and LTP. Previously, we found that Reelin enhances LTP in the adult brain through enhancing NMDAR currents (Weeber et al., 2002; Durakoglugil et al., 2009). Along this line, Reelin treatment for 1 h increases surface expression of GluA1 and GluA2 in the hippocampal CA1 in a PI3K-dependent manner (Qiu et al., 2006a). In this study, we investigated whether Reelin treatment influences mGluR5 agonist DHPG-induced LTD. Extracellular field potentials were recorded from the stratum radiatum. During the entire experiment, hippocampal slices from WT mice were maintained in oxygenated aCSF containing picrotoxin (GABA receptor antagonist, 10 µm) and d-AP5 (NMDAR antagonist, 50 µm). Slices were pretreated with Reelin for 30 min followed by a 10 min treatment with DHPG. After the washout of DHPG, slices were incubated with or without Reelin for an additional 50 min. In 3-month-old mice, Reelin (5 nm) application reduced the long-term LTD response after DHPG (100 µm) treatment (Control 58.16 ± 4.61%, *n* = 14 slices, animals (*a*) = 8 mice vs Reelin-treated slices 84.62 ± 6.86%, *n* = 11 slices, *a* = 6 mice) of baseline (*n* indicates the slice numbers and *a* denotes animal numbers; Fig. 1*A*,*B*), suggesting that Reelin regulates mGluR-LTD.

### Exogenous Reelin reduces STEP_61_ levels and increases GluA2 phosphorylation

Dephosphorylation of the AMPAR subunit GluA2 by tyrosine phosphatases results in AMPAR endocytosis and is essential for DHPG-induced LTD (Moult et al., 2002). DHPG increases the abundance of STEP_61_, a brain-specific phosphatase of GluA2 (Zhang et al., 2008). Thus, to understand how Reelin reduces mGluR-LTD, we examined STEP_61_ levels on Reelin treatment. STEP_61_ protein levels were analyzed in wild-type hippocampal slices after AP-5, picrotoxin, Reelin, and/or DHPG treatment. Mouse hippocampal slices were treated in six-well plates with AP-5, picrotoxin, Reelin, and/or DHPG as described extensively (see above, Materials and Methods). STEP_61_ levels compared with control levels 50 min after DHPG washout were not significantly different (Fig. 1*C*; Control 102.7 ± 3.53% vs post-DHPG: 94.59 ± 12.05%, *n* = 7 experiments, *a* = 7, n.s.). By contrast, in slices pretreated with AP-5, picrotoxin, and Reelin, basal STEP_61_ levels were significantly decreased (Reelin: 65.32 ± 5.72%, *n* = 7 experiments, *a* = 7 animals, *p* = 0.0096). Moreover, we saw a decrease in STEP_61_ in samples cotreated with AP-5, picrotoxin, Reelin, and DHPG (post-DHPG with Reelin: 73.20 ± 5.51%, *n* = 7, *a* = 7, *p* = 0.0482; Fig. 1*C*, 3-month-old mice). These data support the conclusion that Reelin blocks DHPG-induced LTD through STEP_61_.

Activated STEP_61_ dephosphorylates GluA2. Given the observed Reelin-mediated block of DHPG-induced LTD and decreased STEP_61_ levels (Fig. 1 *A*–*C*), we quantified tyrosine phosphorylation (Y869/873/876) of GluA2 after applying DHPG to primary rat cortical neurons (DIV 14) in the absence and presence of AP-5, picrotoxin. and Reelin (Fig. 1*D*). Coapplication of AP-5, picrotoxin, Reelin, and DHPG increased GluA2 phosphorylation (DHPG 100 ± 7.49% vs DHPG + Reelin 124.34 ± 7.16%, *n* = 4 different cultures from four separate rats). Whether Reelin directly increases phosphorylation of GluA2 or indirectly through STEP_61_ still needs to be determined. We propose a model where Reelin blocks mGluR-dependent LTD through increasing the phosphorylation of GluA2 along with a reduction in STEP_61_ levels (Fig. 1*E*).

### mGluR-LTD is occluded in mice lacking Reelin

**Figure 2.**
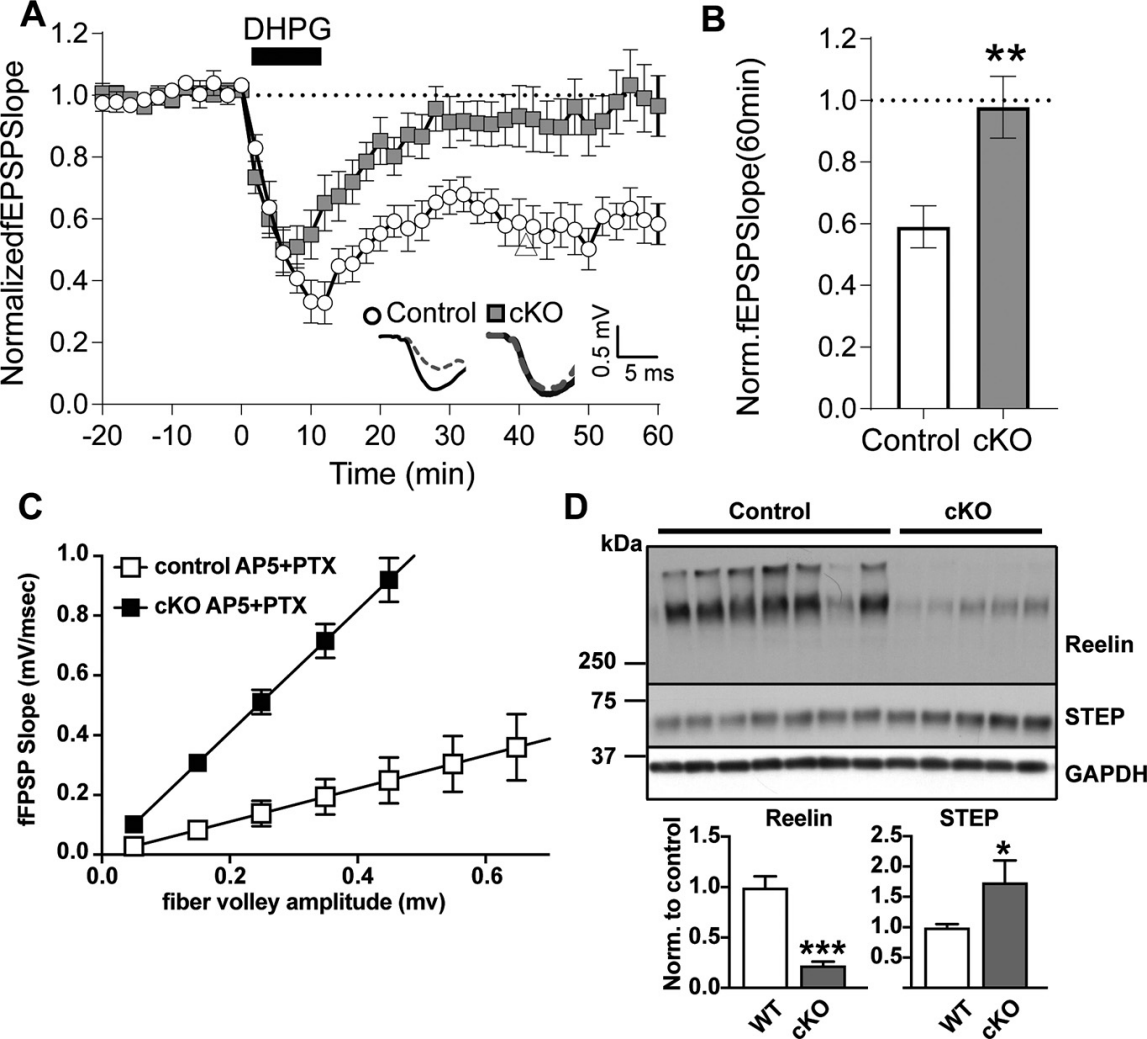
mGluR-LTD is occluded when Reelin is conditionally knocked out. ***A***, Normalized fEPSP slopes before and after DHPG treatment in 3-month-old Reelin cKO and Control slices in CA1 region in the presence of AP-5 (50 μm) and picrotoxin (10 μm). Sample fEPSP traces before and after DHPG (inset, bottom right). (Control, *n* = 11 slices, *a* = 3 animals; cKO, *n* = 13, *a* = 3; two-way RM ANOVA: Reelin-int: *F*_(1,22)_ = 10.68, *p* = 0.0035; Holm–Sidak *post hoc*: Control cKO: 38–60 min, *p* = 0.0441–0.001, time-int: *F*_(40,880)_ = 20.39, *p* < 0.0001; Holm–Sidak *post hoc*: Control: −20 vs 60 min, *p* < 0.0001; cKO: −20 vs 60, *p* > 0.9999). -int, -interaction. ***B***, Reelin knockdown completely blocked DHPG-induced LTD. Quantification of average fEPSP in the last 5 min is shown. Unpaired Student's *t* test was performed on the average of Control slices or Reelin cKO-treated slices 50 min after DHPG treatment (Control *n* = 11, Reelin cKO *n* = 13, *p* = 0.0055). ***C***, Input-output analysis shows increased excitability in the Reelin cKO mice in the presence of AP-5+picrotoxin (filled square). ***D***, Hippocampal protein analysis indicates an increase in STEP_61_ levels in Reelin cKO mice. Top, Confirmation of Reelin conditional knockout efficiency after the injections. (Control, *n* = 7, *a* = 7; Reelin cKO, *n* = 5, *a* = 5; unpaired *t* test, *p* = 0.0002). STEP_61_ levels were measured by immunoblotting of hippocampal lysates from 6-month-old Reelin cKO and tamoxifen-injected Control mice (Control, *n* = 13, *a* = 13; Reelin cKO, *n* = 10, *a* = 10; unpaired *t* test, *p* = 0.0280). Error bars represent the SEM. (*p < 0.05, **p < 0.01).

As exogenous Reelin treatment blocks LTD (Fig. 1*A*,*B*), we next investigated whether depletion of endogenous Reelin has an impact on the same system. Therefore, we induced mGluR-LTD with DHPG (100 µm) in the presence of NMDAR antagonist (AP-5) and GABA receptor antagonist (picrotoxin) in hippocampal slices from 3-month-old conditional Reelin knock-out mice (Reelin cKO), where Reelin was depleted at 4 weeks of age, thereby testing the *in vivo* function of endogenous Reelin in adult mice after the end of the neuronal migration period for which Reelin is required (Herz and Chen, 2006). Intriguingly, we found that in Reelin-deficient slices, DHPG-induced mGluR-LTD was reduced (Control 59.0 ± 6.8%, *n* = 11 slices, *a* = 3 animals; Reelin cKO 97.8 ± 10.0%, *n* = 13, *a* = 3; Fig. 2*A*,*B*). In addition, the input/output curve in the presence of d-AP5 (50 µm) and picrotoxin (10 µm) showed a leftward shift consistent with an increased excitability in the Reelin cKO slices from CA1 (Fig. 2*C*). Considering the spectrum of neuromodulatory roles of Reelin (Wasser and Herz, 2016; Lane-Donovan and Herz, 2017), our findings suggest that Reelin is regulating pathways that are critical for mGluR-mediated LTD.

### Reelin cKO mice have elevated STEP_61_ levels

LTD induction depends on both the phosphorylation and surface expression of GluA2, which is regulated by STEP_61_ levels (Won et al., 2019). We postulated that initial loss of Reelin by tamoxifen-driven Cre-recombination would remove the brake on the LTD suppression, resulting in elevated LTD and increased levels of STEP_61_. To test this hypothesis, we measured hippocampal STEP_61_ levels in 6-month-old Reelin cKO mice. Ablation of Reelin significantly increased STEP_61_ levels (Control 100.0 ± 5.1% vs Reelin cKO 174.5 ± 35.6%; Control *n* = 13, *a* = 13; Reelin cKO *n* = 10, *a* = 10, unpaired *t* test, *p* = 0.0280; Fig. 2*D*). These data, along with the phosphorylation of GluA2 containing AMPARs (Fig. 1*D*), further support a role for Reelin in the regulation of STEP_61._

### Postdevelopmental loss of Reelin increases rectification index in CA1 neurons

**Figure 3.**
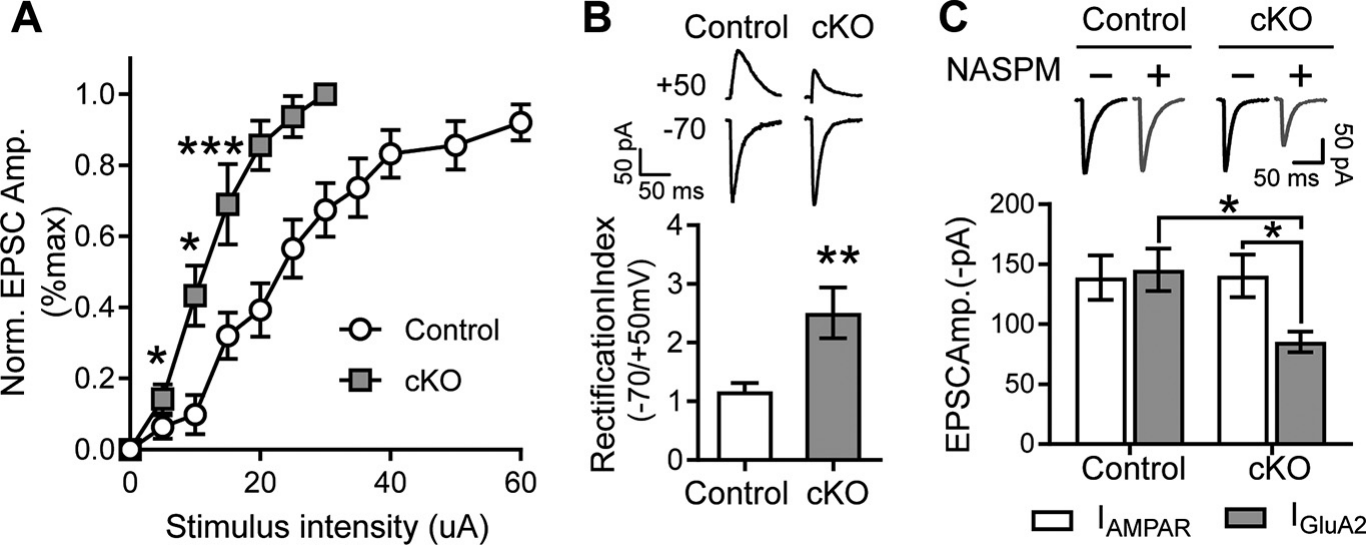
Evoked AMPA single-cell whole-cell currents in the Reelin cKO mice shows the presence of GluA2 lacking AMPA receptors. ***A***, Reelin cKO input-output curve is shifted to the left. Plot of the average AMPAR current output at increasing stimulation intensities depicting enhanced AMPAR currents in Reelin cKO neurons compared with control neurons, indicating Reelin cKO neurons have increased excitability (Control, *n* = 18 cells, *a* = 5; cKO, *n* = 10 cells, *a* = 3; two-way ANOVA: Genotype-int: *F*_(1,108)_ = 12.42, *p* = 0.0006; Sidak's *post hoc*: Control vs cKO: 5–15 uA, *p = 0.0412, ***p = 0.0002, 2- to 3-month-old mice). ***B***, Reelin cKO mice have a higher RI. Reelin cKO mice show higher RI compared with their age-matched controls, which is a strong indication for the presence of GluA2-lacking AMPARs. RI is calculated as the ratio of peaks at −70/+50 mV (Control, *n* = 15 cells, *a* = 11; cKO, *n* = 8 cells, *a* = 5; two-tailed unpaired *t* test: *t*_(21)_ = 3.639, **p = 0.0015, 2- to 3-month-old mice). ***C***, AMPAR currents are more sensitive to PhTXs, specific blockers of GluA2-lacking AMPARs in Reelin cKO mice. Representative EPSC traces (top) and summary plot of the average AMPAR-mediated currents before (I_AMPAR_) and after PhTX (NASPM) application (I_GluA2_; bottom). Pharmacologically isolated evoked AMPAR currents from Reelin cKO mice show higher sensitivity to NASPM (Control, *n* = 6 cells, *a* = 4; cKO, *n* = 7 cells, *a* = 5, two-way RM ANOVA: NASPM-int: *F*_(1,11)_ = 4.207, *p* = 0.0649; Sidak's *post hoc*: I_AMPAR_–I_GluA2_: Control, *p* = 0.9192; cKO, *p = 0.0114, Genotype-int: *F*_(1,11)_2.349, *p* = 0.1536; Sidak's *post hoc*: Control vs cKO: I_AMPAR_, *p* = 0.9973; I_GluA2_, *p = 0.027, 2- to 3-month-old mice). Error bars represent the SEM. (*p < 0.05, **p < 0.01, ***p < 0.001).

During development, lack of Reelin disrupts normal neuronal migration leading to perturbations in lamination of the hippocampus and neocortex. In the adult mice, Reelin can modulate NMDARs and synaptic plasticity (Lane-Donovan et al., 2014, 2015), yet it is not clear how Reelin can regulate glutamatergic receptor surface expression under stress conditions. Because depletion of Reelin after 6 months of age increases STEP_61_ levels, we suspect that downstream targets of STEP_61_ are altered as well. As STEP_61_ regulates surface expression of GluA2-containing AMPARs, we first evaluated the functional properties of evoked AMPA currents using whole-cell recordings in the Reelin cKO mice. We found a significantly greater magnitude of average AMPAR current in CA1 neurons from Reelin cKO mice compared with those from control mice at all stimulus intensities, as seen with a leftward shift of the input-output (IO) curve in the Reelin cKO mice compared with the Controls (Fig. 3*A*; Control, *n* = 18 cells, *a* = 5; Reelin cKO, *n* = 10 cells, *a* = 3). This leftward shift in the IO curve indicates an increase in conductance of AMPARs. Lack of the GluA2 subunit increases the conductance of AMPARs and the ability of polyamines (spermine) to block the receptor at positive holding potentials (Swanson et al., 1997). Thus, we can use the ratio of the AMPAR peak current evoked at −70mV over those evoked at +50mV to calculate the rectification index (RI). An increase in RI would indicate the amount of inwardly rectifying receptors or, as in our case, GluA2-lacking AMPARs. Therefore, we measured the RI in 2- to 3-month-old Reelin cKO mice. Indeed, loss of Reelin increased the RI, suggesting the presence of inwardly rectifying GluA2-lacking AMPARs (Control 1.168 ± 0.14, *n* = 15 cells, *a* = 11 vs Reelin cKO 2.507 ± 0.4, *n* = 8 cells, *a* = 5; Fig. 3*B*). Reelin deficiency increases excitability and RI, which is consistent with a relative lack of GluA2 on the surface.

### Evoked AMPAR currents from Reelin cKO mice have a higher proportion of GluA2-lacking receptors

PhTXs are polyamines that specifically block GluA2-lacking AMPARs. To determine the relative amount of GluA2-lacking AMPARs in the cKO mice, we compared the PhTX sensitivity of AMPAR components of stimulus-evoked excitatory postsynaptic currents (EPSCs) in the CA1 region of 2- to 3-month-old Reelin cKO to the Control mice. The exogenous PhTX (NASPM) induced a robust reduction in AMPAR currents in Reelin cKO (from 140.49 to 85.20 ± 8.66 pA; *n* = 7 cells, *a* = 5), whereas it had no effect on control hippocampi (from 138.99 ± 18.55 to 145.51 ± 17.70 pA; *n* = 6 cells, *a* = 4; Fig. 3*C*), confirming that Reelin cKO mice have an increase in GluA2-lacking AMPARs.

### Spontaneous AMPAR minis have higher amplitude and frequencies in Reelin cKO mice

**Figure 4.**
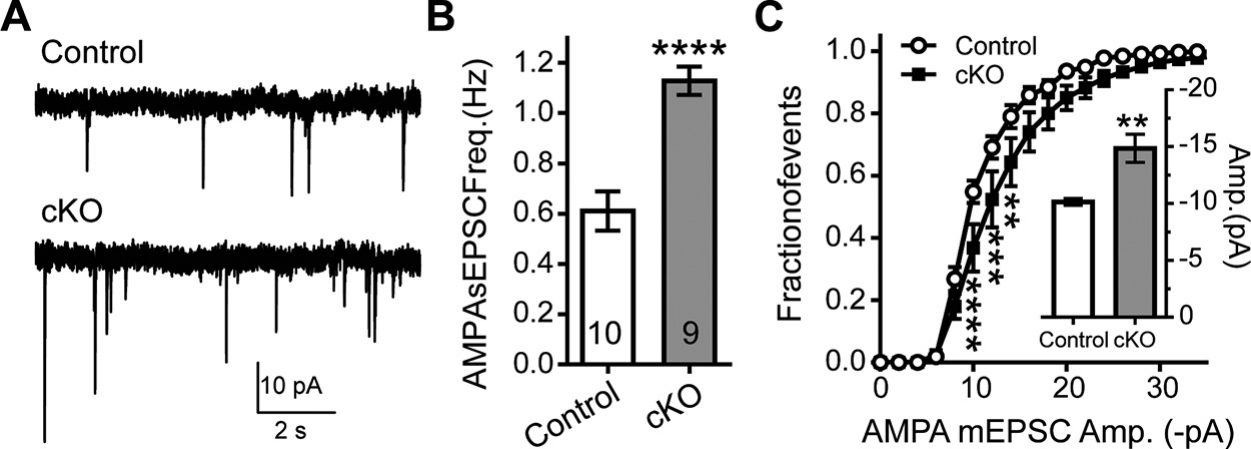
Spontaneous AMPA minis have higher amplitude and frequencies in the Reelin mice. ***A–C***, AMPA currents were pharmacologically isolated with AP-5 (50 μm) and picrotoxin (10 μm). Reelin cKO neurons display increased frequency (Control *n* = 10 cells, *a* = 7 and Reelin cKO *n* = 9 cells, *a* = 5, unpaired *t* test, *p* < 0.001) and amplitude (unpaired *t* test, *p* < 0.001) of spontaneous AMPAR currents compared with WT slices. ***A***, Representative traces. ***B***, Summary plots of frequency analysis (unpaired *t* test, *p* < 0.001). ***C***, Cumulative probability analysis of the amplitudes of AMPAR currents in Reelin cKO and Control CA1 hippocampal neurons shows significance at 10, 12, and 14 pA (two-way RM ANOVA: amplitude × genotype-int *F*_(25,425)_ = 3.023, *p* < 0.0001, amplitude *F*_(25,425)_ = 538.4, *p* < 0.0001, 2- to 3-month-old mice). Error bars represent the SEM. (**p < 0.01, ***p < 0.001,****p < 0.0001).

To further validate if the increased excitability in Reelin cKO mice is mediated by an increase in GluA2-lacking AMPARs, we analyzed spontaneous AMPAR minis, which represent the AMPAR current responses to glutamate release at individual synapses. Reelin cKO neurons display increased amplitude (Control 10.15 ± 0.30 pA, *n* = 10 cells, *a* = 7 vs Reelin cKO 14.84 ± 1.23 pA; *n* = 9 cells, *a* = 5) and frequency (Control 0.61 ± 0.08 Hz vs Reelin cKO 1.128 ± 0.06 Hz) of spontaneous AMPAR currents compared with control neurons (Fig. 4*A–C*). However, both were reduced in Reelin-cKO compared with Control and Reelin-cKO neurons not treated with the inhibitor. The reduced amplitude after NASPM is consistent with an increased number of GluA2-lacking receptors at Reelin-cKO synapses.

### Surface GluA2 expression is significantly reduced in Reelin cKO mice

**Figure 5.**
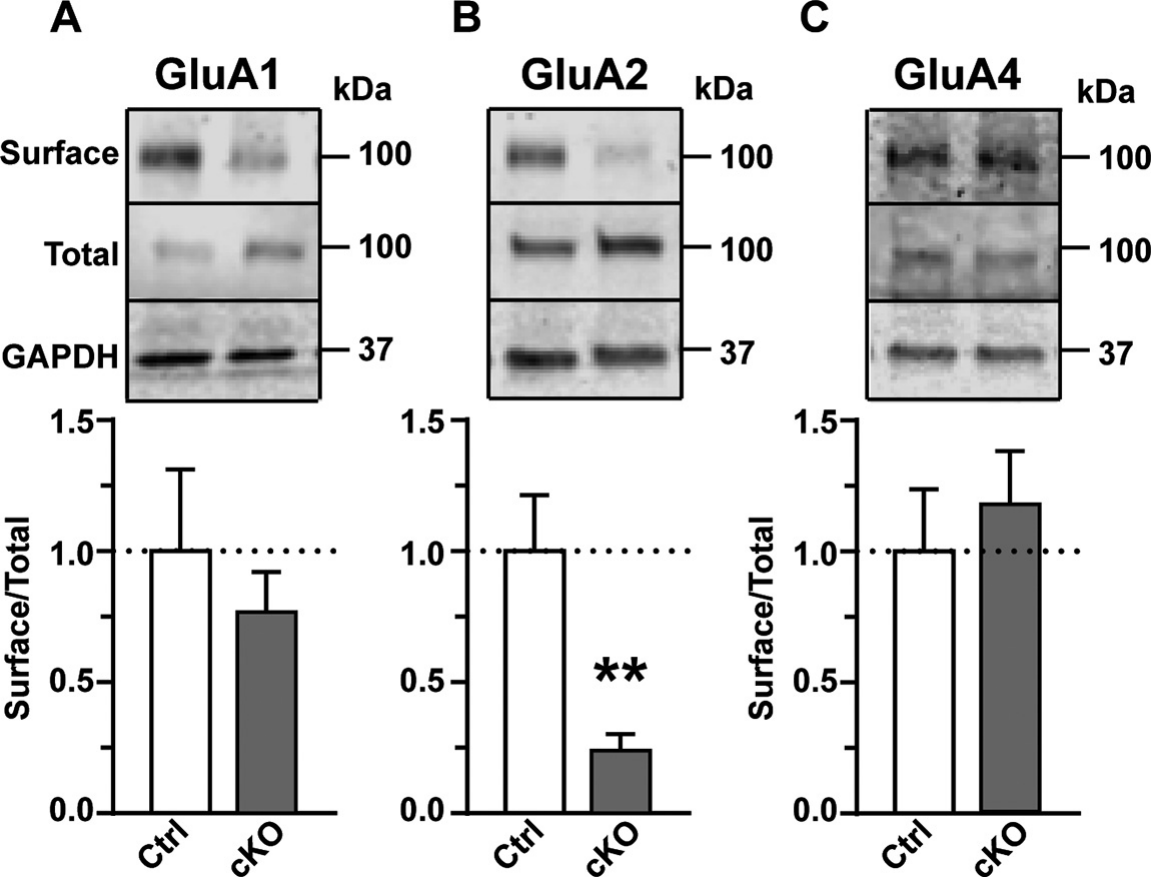
Surface GluA2 expression is significantly reduced in Reelin cKO mice after NMDA receptor block. ***A*–*C***, Slices were treated with AP-5 (50 μm) and picrotoxin (10 μm). Surface to total (S/T) AMPAR subunits (GluA1, GluA2, GluA4) are quantified between Control (Ctrl; *n* = 6, *a* = 6) and Reelin cKO (*n* = 6, *a* = 6) mice at 6 months of age. Top, Representative Western blots of surface and total GluA subunits, GAPDH was used as loading controls. ***A***, For S/T GluA1 no significant difference was observed between Reelin cKO and Control. (*p* = 0.52, *n* = 6, unpaired *t* test). ***B***, The S/T GluA2 level is decreased in Reelin cKO when compared with Control (*p* = 0.007, *n* = 6, unpaired *t* test). ***C***, No significant changes in surface GluA4 were observed in Reelin cKO mice (*p* = 0.23, *n* = 6, unpaired *t* test). Error bars represent the SEM. (**p < 0.01).

The electrophysiology data suggests an increase in GluA2-lacking AMPARs; thus, we next performed surface biotinylation experiments to determine the AMPAR subunits residing on the surface of Reelin cKO neurons. The slices were treated with d-AP5 (50 µm) and picrotoxin (10 µm) to mimic the conditions of the electrophysiological recording experiments. Our results show a significant reduction in surface GluA2 levels (Control 100.0 ± 21.4%, *n* = 6, *a* = 6; Reelin cKO 23.9 ± 6.4%, *n* = 6, *a* = 6), along with a trend to a reduction in surface GluA1 (Control 100.0 ± 31.2%; Reelin cKO 76.7 ± 15.3%) and no significant change in surface GluA4 (Control 100.0 ± 23.9%; Reelin cKO 118.00 ± 20.5), providing further evidence that Reelin depletion in adult mice results in an increase in calcium-permeable GluA2-lacking AMPARs (Fig. 5*A–C*).

## Discussion

**Figure 6.**
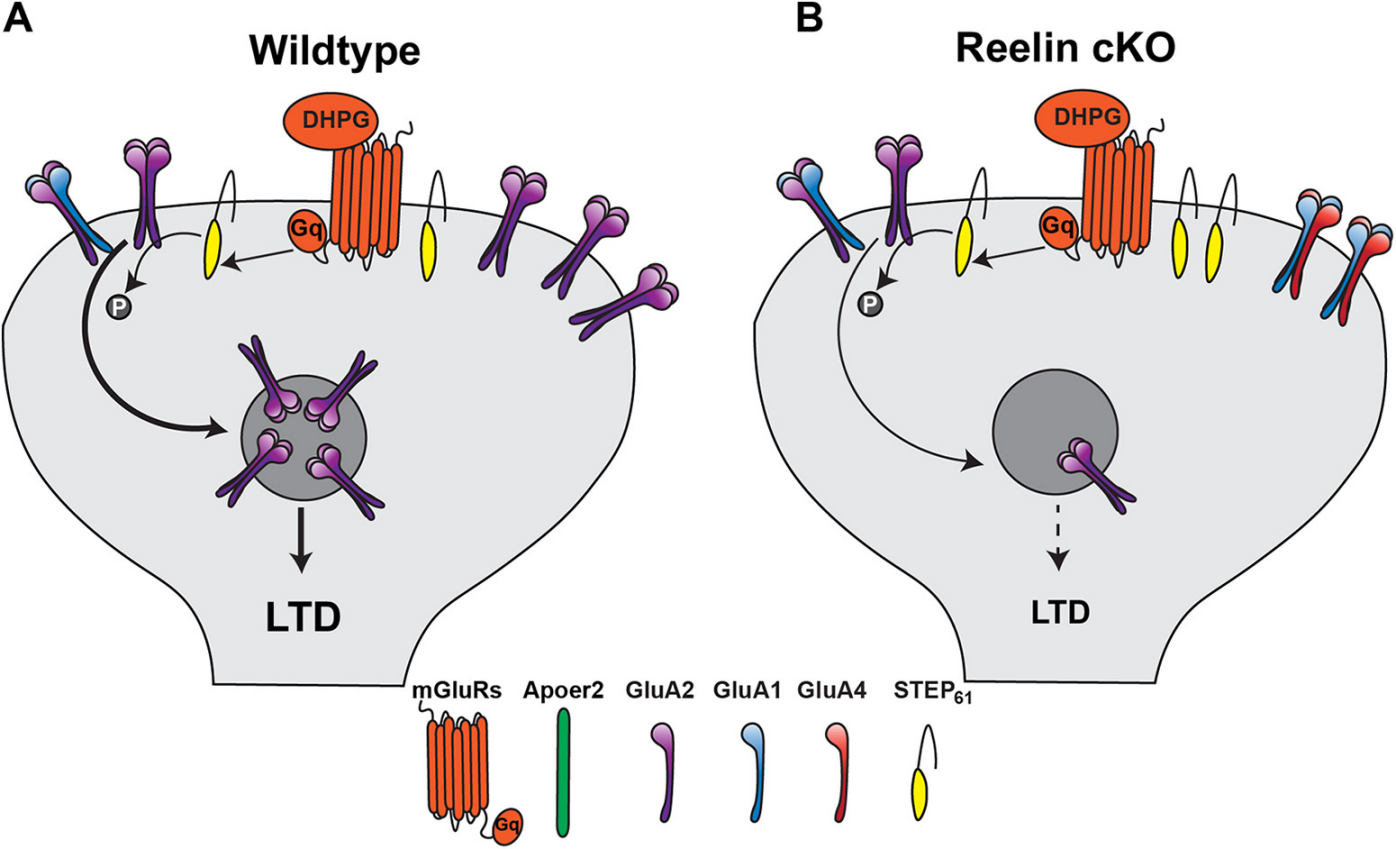
Model for Reelin and STEP_61_ interaction. ***A***, DHPG-induced LTD in wild type. In wild type, synaptic GluA2-containing AMPARs are abundant. DHPG induces mGluR LTD through upregulating STEP_61_ activity. STEP_61_ dephosphorylates GluA2 to induce its endocytosis rendering the synapse less receptive for glutamate and causing LTD. ***B***, Reelin depletion occludes DHPG-induced LTD. When Reelin is knocked out, STEP_61_ levels are elevated. This leads to a chronic dephosphorylation and depletion of GluA2-containing AMPARs from the synaptic surface. In this situation DHPG application can only affect the removal of a small amount of GluA2-containing AMPARs.

We have shown that Reelin inhibits mGluR-LTD, a type of synaptic plasticity that is exacerbated by Aβ in AD mouse models. Specifically, we show that exogenous Reelin reduces STEP_61_ levels. Thus, in the presence of DHPG, Reelin suppresses the dephosphorylation of GluA2 receptors leading to the inhibition of mGluR-LTD (Fig. 6*A*). Conversely, Reelin depletion in the adult mouse brain leads to an increase of STEP_61,_ while causing a reduction in surface expression of GluA2-receptors after NMDAR blockade. Although this finding seems counterintuitive at first, our data suggest that in the Reelin cKO mice, STEP levels are already saturated and GluA2-containing AMPARs are already internalized. Therefore, application of DHPG does not cause further internalization and thus no longer induces LTD (Fig. 6*B*), an effect known as occlusion, which also occurs in amyloid precursor protein and presenilin-1 mice, which can be rescued by repression of the eIF2α kinase PERK (Yang et al., 2016). Importantly there is evidence that ApoE4 treatment in neurons increases excitotoxicity (Buttini et al., 2010). Indeed, our preliminary unpublished data suggest that slices from ApoE4 mice show increased expression of CPARs in an age-dependent manner. This would be consistent with Reelin resistance developing during aging in ApoE4 mice in parallel with ApoE4 mediated risk for AD.

We propose two mechanisms for how Reelin regulates mGluR-LTD: (1) either through directly enhancing GluA2 surface expression and phosphorylation and/or (2) by regulating translation of proteins required for LTD. The first is supported by a study indicating that Reelin enhances the surface expression of AMPARs-containing GluA1 and/or GluA2 (Qiu et al., 2006b). Reelin binding to Apoer2 activates Src family kinases (SFK), which phosphorylate Disabled 1 (Dab1), the first step of Reelin pathway activation. SFKs also phosphorylate GluA2 at residue Y876 (Kohda et al., 2013). Thus, Reelin might have a direct effect on GluA2 phosphorylation through SFKs. Supporting the second mechanism, our current data show that Reelin regulates the protein level of STEP_61_, an important LTD protein. STEP_61_ dephosphorylates tyrosines on GluA2 to induce its endocytosis (Won et al., 2019). Reelin reduces STEP_61_ levels and therefore prevents GluA2 dephosphorylation and endocytosis.

We have shown that exogenous Reelin treatment increases tyrosine (Y869/Y873/876) phosphorylation of GluA2 (Fig. 1*D*), and others showed that Reelin increases GluA2 surface expression (Qiu et al., 2006a; Qiu and Weeber, 2007). The role of Reelin in increasing surface GluA2 and phospho-GluA2 is further supported by decreased surface GluA2 levels we found in Reelin cKO brain slices. It is likely that Reelin controls surface GluA2 through both mechanisms mentioned above. Future studies need to delineate how Reelin regulates STEP_61_ levels. It is possible that Reelin modulates STEP_61_ levels through translational regulation of LTD genes. This model is supported by the finding that Reelin regulates the mTOR and ribosomal protein S6 Kinase (S6K) pathway (Bock et al., 2003; Jossin et al., 2003). S6K regulates translation of LTD proteins like FMRP and STEP_61_. Additionally, our complementary study in Reelin cKO mice shows that STEP_61_ levels are elevated along with a reduction in surface GluA2 receptors (Fig. 5). Consequently, Reelin cKO neurons are unable to respond to DHPG, and LTD is occluded.

The subunit composition of synaptic AMPARs can undergo dynamic changes during physiological as well as pathologic conditions (Weiss, 2011). Physiologically, GluA2-lacking AMPARs are transiently incorporated into the synapse during LTP, which is followed by insertion of GluA2-containing AMPARs that are required for LTP consolidation (Terashima et al., 2008). Pathologically, however, the absence or insufficient expression of GluA2 subunits renders neurons more susceptible to excitotoxicity and neuronal cell death (Liu and Zukin, 2007). Since their discovery, Ca^2+^-permeable AMPARs (CPARs) have been implicated in the pathogenesis of various neurologic conditions, including epilepsy (Egbenya et al., 2018), amyotrophic lateral sclerosis, ischemic insult (Weiss, 2011), and AD (Gaisler-Salomon et al., 2014). Our data support the findings that in the adult mouse brain, Reelin regulates AMPAR subunit composition during neurophysiological and neurodegenerative conditions. In an autosomal dominant form of temporal lobe epilepsy (TLE) patients have increased GluA2-lacking AMPARs (Egbenya et al., 2018). Interestingly, TLE causes neuronal positioning defects in the dentate gyrus of patients because of deficient Reelin expression (Haas et al., 2002; Haas and Frotscher, 2010; Tinnes et al., 2011). Thus, the link between Reelin deficiency and GluA2-lacking AMPARs might be relevant for TLE as well.

GluA2 mRNA is a substrate of adenosine deaminase acting on RNA (ADAR2) and is normally edited by this enzyme to cause a Q to R change at amino acid 607. AMPARs containing the unedited GluA2(Q) subunit are Ca^2+^ permeable, whereas the presence of edited GluA2(R) render the receptor Ca^2+^ impermeable (Swanson et al., 1997). AMPAR-containing GluA2(Q) and GluA2-lacking AMPAR are collectively known as CPARs, and play an important role in synaptic and homeostatic plasticity and in the pathophysiology of various neurologic disorders (Henley and Wilkinson, 2016). Recently, it was found that Reelin-expressing stellate cells of the medial entorhinal cortex are subject to age-dependent GABA receptor subunit α3 editing (Berggaard et al., 2019). Whether Reelin can regulate GluA2 editing remains to be determined.

The N terminus of GluA2 is indispensable for hippocampal mGluR-LTD (Zhou et al., 2011) and depletion of GluA2 ablates mGluR-LTD in adult mice (3 months), but not in younger mice (<1 month), suggesting that GluA2 is involved in mGluR-LTD in an age-dependent manner (Cao et al., 2017, 2018). GluA2-dependent mGluR-LTD in mature mice is mediated through Rho GTPase Rac1 and its downstream actin-regulator cofilin (Zhou et al., 2011). Cofilin phosphorylation is selectively reduced in the mature mouse brain. Conversely, Reelin reduces cofilin activity via phosphorylation through LIMK1 (LIM Domain Kinase 1), a downstream effector of Rac1 (Chai et al., 2009), suggesting that the Reelin/cofilin axis provides another potential LTD-regulating pathway. Thus, it is possible that Reelin is a key regulator in the transition from the juvenile to the mature brain. Future studies are required to dissect these interactions to understand their contributions to overall plasticity and neurodegeneration. Whereas Reelin seems to regulate synaptic plasticity in mature brains dependent on GluA2, other plasticity regulators such as PICK1 (protein interacting with C-Kinase 1) are effective in the juvenile brain independent of GluA2 (Cao et al., 2018)

LTD is induced through activation of Group1 mGluRs (mGluR1 and mGluR5), which induces a cell signaling cascade mediated by the PI3 kinase (PI3K)/Akt and mTOR pathways. This cascade controls the phosphorylation of proteins, for example Fragile X mental retardation protein (FMRP) a critical regulator for the translation of LTD proteins. Phosphorylated FMRP controls protein synthesis by stalling ribosomes on target LTD mRNAs and suppresses the translation of proteins such as STEP_61_, Arc, and MAP1b (Richter and Klann, 2009; Waung and Huber, 2009; Gonatopoulos-Pournatzis et al., 2020). Importantly, and comparable to AD mouse models, FMRP-KO mice have higher STEP_61_ levels (Chatterjee et al., 2018). In this current study, we have shown that Reelin controls STEP_61_ levels, suggesting a role in protein synthesis.

Dysregulation of the mTOR pathway occurs in numerous neurodegenerative and neurodevelopmental diseases including AD and autism (Hoeffer and Klann, 2010; Sharma et al., 2010). Reelin and mTOR signaling pathways share pivotal hub molecules, such as PI3K and Akt. Whereas Reelin acts upstream of PI3K/Akt (Bock et al., 2003), mTOR acts downstream (Khlebodarova et al., 2018). Heterozygous Reelin-KO mice, a genetic model for schizophrenia, have reduced spine density and abnormal fear memory, which is alleviated by a single *in vivo* injection of ketamine or a GluN2B antagonist. The rescue effect by ketamine can be prevented by rapamycin, an inhibitor of the mTOR pathway (Iafrati et al., 2014), further bolstering the role of mTOR in Reelin-related psychiatric and neurodegenerative disorders.

Brain-derived neurotrophic factor (BDNF) is an upstream activator of mTOR during memory formation (Slipczuk et al., 2009). There is evidence for cross-talk between Reelin and BDNF (Ringstedt et al., 1998): BDNF, by binding to its receptors on the Cajal-Retzius cells of the cerebral cortex, downregulates Reelin expression during early postnatal development. In dendrites of cultured neurons, BDNF activates signaling of both 4E-BP and S6K, translational regulators downstream of mTOR (Takei et al., 2001). BDNF promotes proteolysis of PTEN (phosphatase and tensin homolog deleted on chromosome 10), which is a phosphatase that inhibits Akt activity and signaling to mTORC1 and degradation of tuberous sclerosis complex proteins TSC1 and TSC2, both of which are negative regulators of mTORC1 (Ebert and Greenberg, 2013). Along this line, in heterozygous Reeler mice, decreased levels of PTEN were found in the postsynaptic density fractions (Ventruti et al., 2011).

Local protein synthesis and actin remodeling in neurons involves activation of TrkB receptors by the neurotrophin BDNF and Group1 mGluRs by DHPG. As previously described, DHPG increases local translation and tyrosine phosphatase activity of STEP_61_ to dephosphorylate and induce endocytosis of the GluA2-containing AMPARs. Intriguingly, BDNF increases STEP_61_ degradation through PLCγ-dependent proteasomal activation, which requires tyrosine kinase activity (Saavedra et al., 2016). Activation of mGluRs enhances dendritic protein synthesis through mTOR- and ERK-dependent mechanisms. In summary, our data show that Reelin can reduce STEP_61_ translation induced by DHPG application, suggesting a regulatory function at the level of protein synthesis.
